# Effects of hyperventilation on repeated breath-holding while in a fasting state: do risks outweigh the benefits?

**DOI:** 10.1152/ajpregu.00260.2023

**Published:** 2024-02-05

**Authors:** Antonis Elia, Mikael Gennser, Ola Eiken, Michail E. Keramidas

**Affiliations:** Division of Environmental Physiology, Swedish Aerospace Physiology Centre, KTH Royal Institute of Technology, Stockholm, Sweden

**Keywords:** apnea, diving reflex, fasting, hyperventilation, spleen

## Abstract

Breath-holding preceded by either an overnight fast or hyperventilation has been shown to potentiate the risk of a hypoxic blackout. However, no study has explored the combined effects of fasting and hyperventilation on apneic performance and associated physiological responses. Nine nondivers (8 males) attended the laboratory on two separate occasions (≥48 h apart), both after a 12-h overnight fast. During each visit, a hyperoxic rebreathing trial was performed followed by three repeated maximal static apneas preceded by either normal breathing (NORM) or a 30-s hyperventilation (HYPER). Splenic volume, hematology, cardiovascular, and respiratory variables were monitored. There were no interprotocol differences at rest or during hyperoxic rebreathing for any variable (*P* ≥ 0.09). On nine occasions (8 in HYPER), the subjects reached our safety threshold (oxygen saturation 65%) and were asked to abort their apneas, with the preponderance of these incidents (6 of 9) occurring during the third repetition. Across the sequential attempts, longer apneas were recorded in HYPER [median(range), 220(123–324) s vs. 185(78–296) s, *P* ≤ 0.001], with involuntary breathing movements occurring later [134(65–234) s vs. 97(42–200) s, *P* ≤ 0.001] and end-apneic partial end-tidal pressures of oxygen ($\text{P}{\text{ET}}_{{\text{O}}_{2}}$) being lower (*P* ≤ 0.02). During the final repetition, partial end-tidal pressure of carbon dioxide [($\text{P}{\text{ET}}_{{\text{CO}}_{2}}$), 6.53 ± 0.46 kPa vs. 6.01 ± 0.45 kPa, *P* = 0.005] was lower in HYPER. Over the serial attempts, preapneic tidal volume was gradually elevated [from *apnea 1* to *3*, by 0.26 ± 0.24 L (HYPER) and 0.28 ± 0.30 L (NORM), *P* ≤ 0.025], with a correlation noted with preapneic $\text{P}{\text{ET}}_{{\text{CO}}_{2}}$ (*r* = −0.57, *P* < 0.001) and $\text{P}{\text{ET}}_{{\text{O}}_{2}}$ (*r* = 0.76, *P* < 0.001), respectively. In a fasted state, preapnea hyperventilation compared with normal breathing leads to longer apneas but may increase the susceptibility to a hypoxic blackout.

**NEW & NOTEWORTHY** This study shows that breath-holds (apneas) preceded by a 12-h overnight fast coupled with a 30-s hyperventilation as opposed to normal breathing may increase the likelihood of a hypoxic blackout through delaying the excitation of hypercapnic ventilatory sensory chemoreflexes. Evidently, this risk is exacerbated over a series of repeated maximal attempts, possibly due to a shift in preapneic gas tensions facilitated by an unintentional increase in tidal volume breathing.

## INTRODUCTION

As early as 1908, Hill and Flack (1) noted that, in humans, the voluntary apneic breaking point was not solely a function of chemoreflex stress, with subsequent studies highlighting the profound role of volitional factors in dictating ones’ breath-hold duration (2–5). These findings paved the way for dividing a breath-hold in two distinct phases, the easy-going (no immediate urge to breathe) and the struggle (continuously intensified respiratory distress) phases, separated by the so-called physiological breaking point, identified as the point where, in response to ventilatory stimuli reaching a critical threshold, the first involuntary diaphragmatic contraction is registered (6). This locus is of key relevance, since at or close to this point is where nondivers commonly terminate their apneas; an observation that likely relates to a greater reliance on hypercapnic ventilatory sensory chemoreflexes in discerning their volitional breaking point (7, 8). Duly, factors that may contribute toward delaying the physiological breaking point are certainly considered advantageous with respect to apneic performance but equally raise safety concerns.

Breath-hold divers repeatedly flirt with their absolute physiological limits, with arterial partial pressures of oxygen ($\text{P}{\text{a}}_{{\text{O}}_{2}}$) as low as ∼20 mmHg being recorded at breaking point (9); a notable level since it is slightly below the theoretical limit of consciousness (∼27 mmHg) suggested by Nunn (10). It is thus, perhaps, not surprising that hypoxic blackout incidents (i.e., loss of consciousness) are omnipresent within the breath-hold diving community. In light of these recurring episodes, efforts have been made in identifying factors that could potentiate this risk, one of which is preapneic hyperventilation (11, 12). This respiratory maneuver lowers the arterial partial pressure of carbon dioxide ($\text{P}{\text{a}}_{{\text{CO}}_{2}}$) and elevates $\text{P}{\text{a}}_{{\text{O}}_{2}}$, concurrently delaying the excitation of ventilatory sensory chemoreflexes, shifting the physiological breaking point and, resultantly, permitting longer apneas to be attained (13–15). However, loss of consciousness may ensue without forewarning as, by the time the apneist feels impelled to take a breath, $\text{P}{\text{a}}_{{\text{O}}_{2}}$ may fall below its critical level. Even so, and despite numerous efforts made in raising awareness concerning the deleterious effects of hyperventilation (11, 12), this maneuver is still commonly practiced in conjunction with apneic activities.

More recently, dietary intake composition has emerged as yet another component that could predispose apneists to hypoxic blackout (16, 17). The respiratory exchange ratio (RER) produced from the metabolism of carbohydrate, protein, and fat is 1, 0.8, and 0.7, respectively (18, 19). Hence, for a given amount of O_2_ consumed (V̇o_2_), more CO_2_ is produced (V̇co_2_) from the metabolism of carbohydrates than from that of protein or fat. In support of this, we recently demonstrated that, during a hyperoxic rebreathing trial, it took on average 66 s longer for a cohort of healthy nondivers to reach a partial end-tidal CO_2_ pressure ($\text{P}{\text{ET}}_{{\text{CO}}_{2}}$) of 8 kPa when the trial was performed at a lower (0.71 ± 0.08; 162 ± 42 s) than a higher (0.87 ± 0.17; 96 ± 35 s) RER level (17). More importantly, apneas completed in a metabolic state predominated by lipid (i.e., facilitated by a 14-h overnight fast) rather than carbohydrate- or protein turnover, led to significantly longer breath-holds, with these being terminated at progressively lower partial end-tidal O_2_ pressure ($\text{P}{\text{ET}}_{{\text{O}}_{2}}$) but at similar $\text{P}{\text{ET}}_{{\text{CO}}_{2}}$ (17). Altogether, this study reiterated the elemental reliance of nondivers on hypercapnic stimuli in determining their apneic end points but also signified the profound effect of dietary intake composition on apneic performance.

In pursuit of marginal performance gains, during competitions as well as training sessions, it is becoming increasingly evident that most athletes opt to fast and hyperventilate before their maximal attempts (20). Although the initial reduction in $\text{P}{\text{a}}_{{\text{CO}}_{2}}$ as well as the attenuated rate of its accumulation facilitated by this amalgamation will certainly confer advantages, the associated risks remain largely unknown. In this regard, it is presently unclear whether, and to what extent, performing a series of repeated maximal apneas after a brief hyperventilation period in a fasting state could influence the magnitude of the apnea-induced physiological responses and/or further exacerbate the risk of a hypoxic blackout. Considering that a leading cause of death when engaging in breath-hold-related activities is drowning initiated by hypoxia (21) and given an ever-increasing number of people taking up this activity as recreational and/or professional sport, enhancing our understanding of factors that could potentiate this risk is peremptory from a safety point of view.

Thence this study aimed to investigate the effect of fasting with or without preapneic hyperventilation on apneic performance and associated physiological responses over a series of repeated breath-holds. It was hypothesized that apneas performed following a short hyperventilation period as opposed to after normal breathing would improve apneic performance but would lead to greater desaturation levels consequently exacerbating the risk of hypoxic blackout.

## MATERIALS AND METHODS

### Ethics Approval

Ethics approval for this study was granted by the Swedish Ethical Review Authority (Approval No: 2022-02278), and all experimental procedures were performed with the standards set by the latest revision of the Declaration of Helsinki, except for the registration in a database.

Before the onset of the experimental sessions, potential subjects underwent a physical examination by a physician, with only individuals who satisfied the inclusion criteria with a clean health record (i.e., no history of cardiorespiratory disorders nor any other health conditions such as epilepsy or diabetes) being included in the study. In addition, subjects were briefed in detail about the purpose of the study, the experimental procedures, and the potential risks and benefits, before giving their written consent.

### Subjects

Nine, nonsmoking, healthy adults (8 males) volunteered to participate in this study [mean ± standard deviation (SD); age, 29 ± 12 yr; body mass, 79 ± 7 kg; height, 1.8 ± 0.1 m; body mass index, 24 ± 3 kg/m^2^].

### Familiarization Session

Approximately a week before the experimental procedures, subjects underwent a familiarization session that introduced them to the trial conditions, preapneic breathing protocols, requirements, testing environment, and equipment. Moreover, a practice hyperoxic rebreathing trial was performed to ensure that the subjects understood and were familiarized with the protocol.

#### Twenty-four hour dietary recall.

Subjects were instructed to record their dietary intake for 24 h preceding the 12-h fasting protocol. Each subject was provided with an open-ended 24-h dietary recall diary; was then briefed on how to record the dietary intake and was provided with written examples for future reference. During the 24-h dietary recall period, the subjects were instructed to report all consumed foods and beverages by documenting the following: *1*) time of consumption, *2*) estimated consumed quantity expressed as a standard volume/weight, and if present, *3*) brand name. Separate forms were included to report on homemade recipes so that the name of the dish, the total quantity of each ingredient used, and the fraction of dish consumed could be stated. Moreover, if subjects opted to consume a ready-made meal, they were encouraged to take a photo of the nutrition fact food label.

### Experimental Protocol

All experimental procedures were conducted at the same time of the day (i.e., morning) on two separate days (i.e., interspersed ≥ 48-h) at the Division of Environmental Physiology of the KTH Royal Institute of Technology. During each testing day, subjects were instructed to report to the laboratory following a 12-h overnight fast and a minimum of 14-h and 48-h abstinence from caffeine- and alcohol-containing beverages, respectively. In addition, subjects were asked to refrain from strenuous physical activity for 24-h before and during each testing day.

Before each experimental session, the gas analyzers (Datex Normocap 200, Datex·Ohmeda, Helsinki, Finland) for assessing $\text{P}{\text{ET}}_{{\text{O}}_{2}}$ and $\text{P}{\text{ET}}_{{\text{CO}}_{2}}$ were calibrated with known gas mixtures. Specifically, before the resting pulmonary gas exchange test, the O_2_ analyzer was calibrated with room-air (20.93% O_2_) and a 16% O_2_ mixture, and the CO_2_ analyzer with room air (0.04% CO_2_) and a 5% CO_2_ mixture. Likewise, before the hyperoxic rebreathing and apnea trials, the O_2_ analyzer was calibrated with nitrogen (100% N_2_; 0% O_2_), room air (20.93% O_2_), and a 30% O_2_ mixture, and the CO_2_ analyzer was calibrated with room air (0.04% CO_2_) and an 8% CO_2_ mixture. All gas volumes were converted to standard temperature, pressure, and dry conditions. In addition, all cardiopulmonary data were recorded at 20 Hz in a computer-based system and were analyzed using the AcqKnowledge 3.9.3 software (Biopac Systems, MP100A-CE).

#### Arterial pressures and cardiovascular variables.

Beat-to-beat systolic arterial pressure (SAP), diastolic arterial pressure (DAP), and mean arterial pressure (MAP) were measured continuously using a volume-clamp technique (Finometer, Finapres Medical Systems BV, Amsterdam, The Netherlands), with the pressure cuff placed around the middle phalanx of the left middle finger, and with the reference pressure transducer positioned at the level of the heart. Heart rate was derived from the arterial pressure curves as the inverse of the interbeat interval.

Stroke volume (SV) was calculated based on the obtained arterial pulse waveform using the Modelflow method (22), which incorporates age, height, and body mass, and simulates aortic flow waveforms from an arterial pressure signal using a nonlinear three-element model of the aortic input impedance (Finometer, Finapres Medical Systems BV, Amsterdam, The Netherlands). Cardiac output (CO) was computed as SV multiplied by heart rate, and total peripheral resistance (TPR) was calculated as the quotient of MAP and CO.

### Baseline Measurements

**Figure 1.**
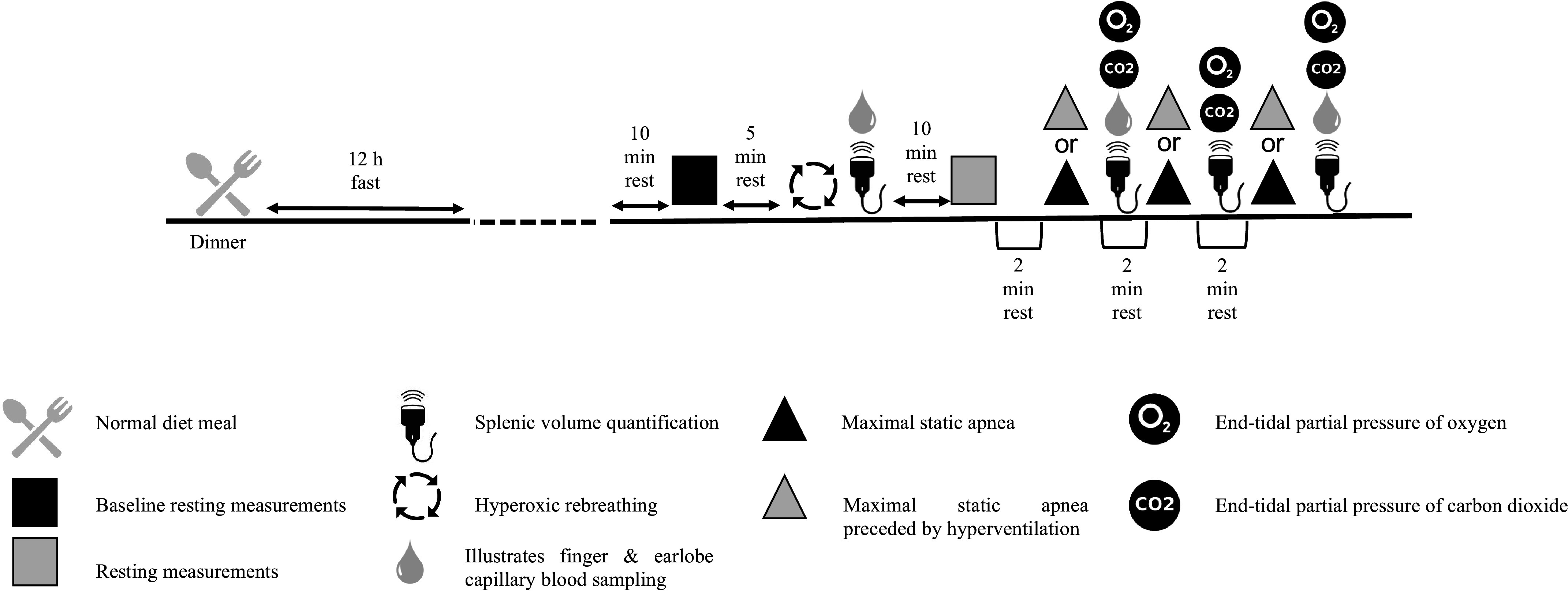
Schematic representation depicting the experimental design and the data collection time points.

After their arrival at the laboratory, the subjects’ anthropometric characteristics were evaluated, including body weight and height (Vetek, Väddö, Sweden). Subjects then underwent a 10-min seated rest followed by measurement of their arterial pressures, CO, SV, TPR, heart rate, and earlobe peripheral oxyhemoglobin saturation ($\text{S}{\text{p}}_{{\text{O}}_{2}}$) levels (Radical-7, Masimo, Irvine, CA; Fig. 1). The subjects’ splenic volumes were then quantified by a noninvasive ultrasonic portable device (Philips CX50, The Netherlands) using a technique described previously (23, 24). Briefly, subjects were seated upright while the site for spleen measurements was identified from the dorsal side. Thereafter, three measurements of each triaxial measurement point of the spleen’s maximal length (L), thickness (T), and width (W) were determined, with the mean for each point being used to calculate splenic volume using the Pilström formula [Lπ (WT − T^2^)/3] [coefficient of variation (CV) ∼ 6%]. Finger capillary blood samples were then collected to assess hematocrit (Thermo Scientific Pico 21 Microcentrifuge; Waltham, MA) and the concentration of glucose (Accu-Check, Aviva, Mannheim, Germany), and an earlobe capillary blood sample was collected to quantify hemoglobin concentration (HemoCue Hb 201+ DM System, Ängelholm, Sweden).

#### Pulmonary gas exchange.

In a seated position, subjects were instrumented with a facemask and breathed through a low resistance, nonreturn two-way valve (Hans Rudolf, MO), with the inspiratory volume being measured continuously with a turbine ventilation module (KL Engineering, Northridge, CA). The expired air was collected via a hose in a 10-L Plexiglas mixing box, from which samples were drawn continuously for analyses of the pressure of O_2_ (Po_2_; using a paramagnetic sensor) and CO_2_ (Pco_2_) (with an infrared analyzer; Datex Normocap 200, Datex·Ohmeda, Helsinki, Finland). After the subjects adjusted to the facemask and baseline values had been stable for 10 min (variability in resting measurements ∼5%), the inspired gas volume (i.e., corrected for temperature and humidity) and mean O_2_ and CO_2_ levels (i.e., derived from a 7-min period) were used to calculate minute ventilation (V̇i), V̇o_2_, V̇co_2_, and RER, with values being expressed at standard temperature and pressure and dry conditions.

#### Hyperoxic rebreathing.

The rebreathing apparatus incorporated a mouthpiece, nose-clip, turbine ventilation module, and a three-way valve to allow switching the airflow between room air and a 6-L latex anesthetic bag prefilled with 95.4% O_2_ and 4.6% CO_2_. A sample flow of 90 mL/min from the mouthpiece (Gas Sample Line 1 M, Datex·Ohmeda, Helsinki, Finland) permitted continuous analysis of $\text{P}{\text{ET}}_{{\text{CO}}_{2}}$ and $\text{P}{\text{ET}}_{{\text{O}}_{2}}$ (Datex Normocap 200, Datex·Ohmeda, Helsinki, Finland) with the sampled gas being continually redirected back to the rebreathing bag to ensure the maintenance of a closed system. Throughout the rebreathing trial, the subjects’ $\text{P}{\text{ET}}_{{\text{CO}}_{2}}$, $\text{P}{\text{ET}}_{{\text{O}}_{2}}$, V̇i, breathing-frequency (*f*_B_), $\text{S}{\text{p}}_{{\text{O}}_{2}}$, and heart rate were recorded continuously (Biopac Systems, MP100A-CE).

Before the rebreathing trial was commenced, subjects completed a 5-min seated rest period while breathing room air (Fig. 1). Subsequently, they performed a deep exhalation and were switched to the rebreathing circuit, whereupon they inspired three large breaths to equilibrate with the circuit. The subjects were then instructed to continue breathing normally (i.e., as they spontaneously felt the urge to) until one of the following protocol termination criteria was met: *1*) $\text{P}{\text{ET}}_{{\text{CO}}_{2}}$ of 8 kPa was reached; *2*) the subjects signaled their level of tolerance; or *3*) the rebreathing bag was emptied. Once switched back to room air, the subjects’ splenic volume, hematocrit, concentrations of hemoglobin, and blood glucose were quantified (Fig. 1).

After the rebreathing trial, subjects underwent a 10-min seated rest period to allow for adequate recovery to restore baseline (Fig. 1). During this period the subjects were allowed to relax and breathe normally (room air) followed by assessment of their resting $\text{P}{\text{ET}}_{{\text{CO}}_{2}}$, $\text{P}{\text{ET}}_{{\text{O}}_{2}}$, tidal volume (V_T_), hemodynamics, $\text{S}{\text{p}}_{{\text{O}}_{2}}$, splenic volume, and hematology (Fig. 1).

#### Static apneas.

The apneic protocol composed of a series of three repeated maximal static apneas interspersed by 2-min resting periods. Throughout this protocol, the subject’s cardiac responses, arterial pressures, and $\text{S}{\text{p}}_{{\text{O}}_{2}}$ were monitored continuously, whereas *f*_B_, V_T_, $\text{P}{\text{ET}}_{{\text{O}}_{2}}$, and $\text{P}{\text{ET}}_{{\text{O}}_{2}}$ were continually assessed before and immediately after each successive attempt (Fig. 1).

In a seated position, the subjects underwent a 2-min resting period in which they were allowed to relax and breathe normally before commencing their maximal attempts. A 1-min warning was provided and thereon at 30 s the subjects either continued breathing normally (NORM) or were instructed to hyperventilate (HYPER), with the latter being dictated by a metronome (rate of breathing); in both protocols “lung packing” was prohibited. A 10-s countdown was provided with each apnea commencing after a deep inspiration (i.e., at near vital capacity; 25). At completion of each apnea, their splenic volume was assessed, whereas the hemoglobin was evaluated after the first and third bout (Fig. 1). This procedure was repeated overall three times during each experimental visit.

The order of the preapneic breathing protocols was randomized, with five and four subjects performing first the NORM and HYPER, respectively.

### Data and Statistical Analyses

Temporal changes in hemodynamics were plotted and were used to graphically divide each breath-hold into three distinct phases, according to the method of Perini et al. (26). Briefly, *phase I* (*PhI*) was defined as the “transition phase” occurring between the immediate start of the breath-hold until a steady state was reached in SAP, DAP, and HR where *phase II* (*PhII*) ensued. *Phase III* (*PhIII*) accounted for the point where the steady state was interrupted and a continuous rise in SAP and DAP was exhibited until the apneic end point.

All data were statistically analyzed using SPSS Statistics software version 26 (International Business Machines, Corp., Armonk, NY). The Shapiro–Wilk test was used to assess whether data were normally distributed. Sphericity was assessed using Mauchly’s test of sphericity; where the assumption of sphericity was violated, the Greenhouse–Geisser correction was applied. Paired sample *t* tests were used to assess for differences between the baseline measurements collected before the hyperoxic rebreathing and apneic trials. The ventilatory reactivity data from the rebreathing trials were plotted for inspired minute ventilation (V̇i/$\text{P}{\text{ET}}_{{\text{CO}}_{2}}$), and linear regression was performed. The slope constant derived from the slopes of the individual plots was then compared using paired sample *t* tests. A one-way analysis of variance (ANOVA) was used to assess for differences between variables collated before and after the hyperoxic rebreathing for each protocol. A two-way repeated-measures ANOVA was used to assess for differences from resting baseline/preapneic levels and between protocols for the apneic trials. Time spent in each phase (*PhI*, *PhII*, and *PhIII*) was evaluated using a two-way repeated-measured ANOVA. Where significant differences were detected in the ANOVA tests, the post hoc Bonferroni test was utilized for contrast comparisons. Pearson correlation was used to assess the relationship between the onset of involuntary breathing movement (IBM) (i.e., the time from start of apnea to the first IBM manifestation) and *phase III* (i.e., the time to the start of *PhIII*), and to examine the relationship between preapneic V_T_ and $\text{P}{\text{ET}}_{{\text{O}}_{2}}$ and $\text{P}{\text{ET}}_{{\text{CO}}_{2}}$ levels. Data are reported as means ± SD, and significance was accepted at *P* < 0.05. Exact *P* values for single comparisons are reported down to *P* = 0.001; smaller values are reported as *P* < 0.001. The signs ≥ and ≤ are used to denote the smallest/biggest *P* value of several. GraphPad Prism version 7.0c (GraphPad Software, Inc., La Jolla, CA) was used to construct figures.

## RESULTS

All subjects completed the experimental trials successfully without any hypoxic blackout incident. However, on nine occasions (1 in NORM; 8 in HYPER), five subjects were asked to abort their attempts due to reaching our safety threshold ($\text{S}{\text{p}}_{{\text{O}}_{2}}$ ∼ 65%). Specifically, the preponderance of these incidents (6 of 9) occurred during the third repetition (1 in NORM; 5 in HYPER), with the rest being seldomly recorded in the earlier attempts (HYPER; 1 in first repetition; 2 in second repetition).

### Baseline Measurements

At baseline, no differences were denoted between the two experimental trials for any variable (*P* ≥ 0.093; Table 1). Likewise, there were no differences when resting measurements collected before the hyperoxic rebreathing trials (Table 1) were compared with those gathered before the apneic trials (see Table 3) in splenic volume (*P* ≥ 0.192) or $\text{S}{\text{p}}_{{\text{O}}_{2}}$ (*P* = 1), or in cardiovascular (*P* ≥ 0.095) or hematological (*P* ≥ 0.075) variables.

**Table 1.** Resting cardiorespiratory, hematological, and splenic volume characteristics for each experimental session

Variables	Normal	Hyper
SAP, mmHg	127 ± 10	131 ± 11
DAP, mmHg	79 ± 9	81 ± 6
MAP, mmHg	97 ± 11	100 ± 7
HR, beats/min	71 ± 12	67 ± 11
$\text{S}{\text{p}}_{{\text{O}}_{2}}$, %	98 ± 1	98 ± 1
SV, mL/beat	82 ± 15	80 ± 14
CO, L/min	5.8 ± 1.7	5.3 ± 1.3
TPR, mmHg·min·L^−1^	18.2 ± 5.8	19.9 ± 6.4
V̇o_2_, L/min	0.31 ± 0.05	0.29 ± 0.05
V̇co_2_, L/min	0.24 ± 0.04	0.23 ± 0.03
RER	0.77 ± 0.05	0.79 ± 0.11
Spleen, mL	273 ± 55	258 ± 39
Blood glucose, mmol/L	6.4 ± 0.5	5.7 ± 0.7
Hematocrit, %	45 ± 4	44 ± 4
Hemoglobin, g/L	158 ± 10	153 ± 9

Data are means ± SD. CO, cardiac output; DAP, diastolic arterial pressure; HR, heart rate; MAP, mean arterial pressure; RER, respiratory exchange ratio; SAP, systolic arterial pressure; $\text{S}{\text{p}}_{{\text{O}}_{2}}$, peripheral oxyhemoglobin; SV, stroke volume; TPR, total peripheral resistance; V̇o_2_, volume of oxygen; V̇co_2_, volume of carbon dioxide.

### Hyperoxic Rebreathing

A similar ventilatory reactivity was detected when the constant of proportionality obtained from the slopes of the two trials was compared (NORM, 11.1 ± 6.3; HYPER, 10.8 ± 8.2, *P* = 0.974). Likewise, the time taken to reach the end point $\text{P}{\text{ET}}_{{\text{CO}}_{2}}$ of 8 kPa was similar in the two sessions (NORM, 150 ± 31 s; HYPER, 152 ± 28 s, *P* = 0.910).

A splenic contraction was noted immediately after the hyperoxic rebreathing trials (*P* < 0.001). Specifically, splenic volume was reduced by 24 ± 9 mL (*P* < 0.001) and 25 ± 17 mL (*P* = 0.012) in NORM and HYPER, respectively. No intrasession differences were documented in any of the hematological variables nor in $\text{S}{\text{p}}_{{\text{O}}_{2}}$ (*P* ≥ 0.292). Likewise, there were no between protocol differences in splenic volumes, hematology, and $\text{S}{\text{p}}_{{\text{O}}_{2}}$ (*P* ≥ 0.129).

### Apneas

#### Duration.

**Figure 2.**
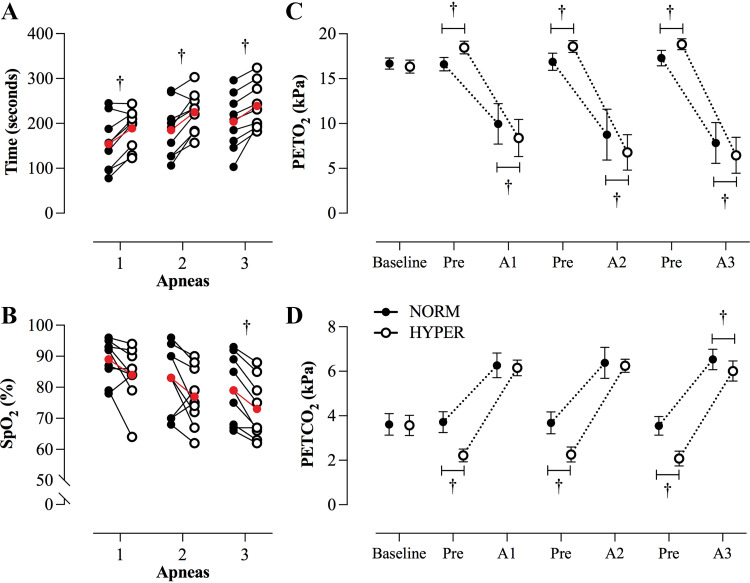
Individual (white and black dots) and mean (red dots) apneic durations (*A*) and breaking point $\text{S}{\text{p}}_{{\text{O}}_{2}}$ levels (*B*) for each repeated attempt in both protocols. *C* and *D*: mean (± SD) $\text{P}{\text{ET}}_{{\text{O}}_{2}}$ and $\text{P}{\text{ET}}_{{\text{CO}}_{2}}$ levels at baseline, before, and at the end of each successive apneic attempt for both protocols. †Significance of between-protocol differences (*P* < 0.05) are presented. Data were analyzed with a two-way repeated measures analysis of variance, followed by Bonferroni post hoc test. HYPER, hyperventilation; NORM, normal breathing; $\text{P}{\text{ET}}_{{\text{O}}_{2}}$, partial end-tidal pressure of oxygen; $\text{P}{\text{ET}}_{{\text{CO}}_{2}}$, partial end-tidal pressure of carbon dioxide; SD, standard deviation; $\text{S}{\text{p}}_{{\text{O}}_{2}}$, peripheral oxygen saturation.

During both experimental trials, subjects attained progressively longer breath-holds across their successive attempts (*P* < 0.001), with invariably longer apneas in HYPER than in NORM (*P* ≤ 0.008; Table 3, Fig. 2*A*).

#### Peripheral oxyhemoglobin saturation.

A reduction in $\text{S}{\text{p}}_{{\text{O}}_{2}}$ was recorded from basal levels during each sequential maximal apneic repetition in both experimental trials (*P* < 0.001; Table 3). Notably, a significant difference was identified between protocols both at breaking point (*P* = 0.020) and nadir (*P* = 0.010). At breaking point, $\text{S}{\text{p}}_{{\text{O}}_{2}}$ was lower in HYPER only during the third apneic attempt (*P* < 0.001; Fig. 2*B*), whereas, with regard to the nadir values, a more pronounced $\text{S}{\text{p}}_{{\text{O}}_{2}}$ reduction in HYPER than in NORM both after the second (*P* = 0.045) and third bout (*P* = 0.002) (Table 3).

#### Cardiovascular responses.

At breaking point, SAP, DAP, MAP, and TPR were higher than baseline (*P* ≤ 0.005) during both experimental trials, whereas no differences were detected in HR, SV, and CO (*P* ≥ 0.372). For all cardiovascular variables, postapneic values were comparable between protocols (*P* ≥ 0.146) (Table 3).

*Phase I* and *phase II* were recognized in all subjects, lasting, respectively, 20 ± 5 s and 101 ± 42 s in NORM, and 25 ± 6 s and 132 ± 36 s in HYPER, whereas *phase III* was only documented in eight of nine subjects (NORM, 72 ± 32 s; HYPER, 65 ± 30 s). No differences were identified in *phase I* and *III* over the series of repeated bouts (*P* ≥ 0.112) nor between protocols (*P* ≥ 0.097; Table 3). In contrast, *phase II* was crescively extended across the succeeding apneas (*P* < 0.001), with it being significantly longer in HYPER than in NORM (*P* < 0.001; Table 3).

#### Involuntary breathing movements.

IBMs were registered in all subjects across the apneic repetitions and protocols. HYPER delayed the manifestation of IBMs by ∼45 s (*P* ≤ 0.005) during each successive apnea compared with NORM (Table 3). In addition, a positive moderate correlation (*r* = 0.68, *R*^2^ = 0.47, *P* < 0.001) was noted between the onset of the IBM and *phase III*.

#### Pet_O_*2*__ and Pet_CO_*2*__.

Regardless of the protocol, postapneic $\text{P}{\text{ET}}_{{\text{O}}_{2}}$ levels were lower and $\text{P}{\text{ET}}_{{\text{CO}}_{2}}$ levels were higher than the preapneic values in all apnea bouts (*P* ≤ 0.001; Table 2).

**Table 2.** Partial end-tidal pressures of oxygen and carbon dioxide pre and post the repeated maximal static apneic attempts

		*Apnea 1*			*Apnea 2*			*Apnea 3*		
Protocol	Baseline	Pre	End	Δ	Pre	End	Δ	Pre	End	Δ
$P{\mathrm{ET}}_{O_{2}}$, *kPa*										
NORM	16.68 ± 0.63	16.61 ± 0.74	9.95 ± 2.25*	−6.66 ± 2.75	16.88 ± 0.96	8.76 ± 2.84*	−8.12 ± 3.57	17.30 ± 0.86‡#	7.83 ± 2.28*	−9.47 ± 2.96
HYPER	16.34 ± 0.73	18.47 ± 0.71†	8.38 ± 2.08†*	−10.09 ± 2.26†	18.58 ± 0.66†	6.78 ± 1.97†*	−11.80 ± 1.90†	18.83 ± 0.61†‡	6.46 ± 2.00†*	−12.37 ± 1.80†
$P{\mathrm{ET}}_{{\mathrm{CO}}_{2}}$, *kPa*										
NORM	3.61 ± 0.48	3.71 ± 0.47	6.27 ± 0.55*	+2.55 ± 0.61	3.68 ± 0.49	6.38 ± 0.70*	+2.70 ± 0.70	3.55 ± 0.42	6.53 ± 0.46*	+2.99 ± 0.46
HYPER	3.57 ± 0.45	2.22 ± 0.29†	6.15 ± 0.35*	+3.93 ± 0.36†	2.26 ± 0.34†	6.25 ± 0.30*	+3.99 ± 0.22†	2.08 ± 0.33†#	6.01 ± 0.45†*	+3.93 ± 0.41†

Data are means ± SD. Data were analyzed with a two-way repeated measures ANOVA, followed by Bonferroni post hoc test (*P* < 0.05). Significant (*P* < 0.05) difference from *pre vs. end, †between protocol differences, ‡differences from *preapnea 1* vs. *2* or *3*, #difference from *preapnea 2* vs. *3* are signified. Δ, delta difference of pre minus end; HYPER, hyperventilation; NORM, normal breathing; $\text{P}{\text{ET}}_{{\text{O}}_{2}}$, partial end-tidal pressure of oxygen; $\text{P}{\text{ET}}_{{\text{CO}}_{2}}$, partial end-tidal pressure of carbon dioxide.

In HYPER, subjects commenced their maximal efforts at higher $\text{P}{\text{ET}}_{{\text{O}}_{2}}$ (*P* < 0.001) and lower $\text{P}{\text{ET}}_{{\text{CO}}_{2}}$ (*P* ≤ 0.004) than NORM (Table 2), with their apneic attempts being terminated at significantly lower $\text{P}{\text{ET}}_{{\text{O}}_{2}}$ (*P* < 0.001; Fig. 2, *C–D*). A lower $\text{P}{\text{ET}}_{{\text{CO}}_{2}}$ (*P* = 0.005) was discerned after the third apneic repetition in HYPER than in NORM (Table 2; Fig. 2*D*), whereas no interprotocol differences were noted in the preceding attempts (*P* ≥ 0.583).

#### Breathing frequency and tidal volume.

In NORM, during the 30-s breathing intervention, no differences were noted in *f*_B_ across the serial attempts (*preapnea 1*, 5 ± 1; *preapnea 2*, 6 ± 1; *preapnea 3*, 6 ± 1).

**Figure 3.**
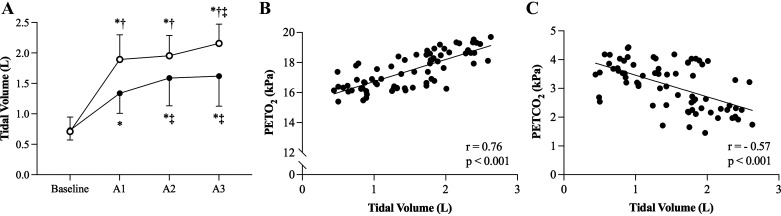
Mean (± SD) tidal volume at baseline and before each maximal apneic attempt in NORM (closed circles, black) and HYPER (open circles, white) (*A*). Relationship between preapneic tidal volume and $\text{P}{\text{ET}}_{{\text{O}}_{2}}$ (*B*) and $\text{P}{\text{ET}}_{{\text{CO}}_{2}}$ (*C*) for both apneic protocols combined. Data were analyzed with a two-way repeated measures analysis of variance, followed by Bonferroni post hoc test (*A*). A Pearson correlation coefficient test was used to assess for linearity between variables (*B* and *C*). *Significant difference (*P* < 0.05) compared with baseline, †significant (*P* < 0.05) between protocol differences; ‡significant difference (*P* < 0.05) from *apnea 1* vs. *apnea 2* or *3*. HYPER, hyperventilation; NORM, normal breathing; $\text{P}{\text{ET}}_{{\text{O}}_{2}}$, partial end-tidal pressure of oxygen; $\text{P}{\text{ET}}_{{\text{CO}}_{2}}$, partial end-tidal pressure of carbon dioxide; SD, standard deviation.

At baseline, resting V_T_ was on average 0.73 ± 0.22 L and 0.71 ± 0.14 L in NORM and HYPER, respectively. We recorded a progressive increase in V_T_ across the apneic trials (*P* < 0.001; Fig. 3*A*). Specifically, over the 30-s breathing interventions V_T_ increased from 1.34 ± 0.44 L and 1.89 ± 0.41 L during the first apnea, to 1.59 ± 0.46 L (*P* = 0.029) and 1.95 ± 0.33 L (*P* = 0.877) in the second, and to 1.62 ± 0.49 L (*P* = 0.012) and 2.16 ± 0.32 L (*P* = 0.021) in the third apnea in NORM and HYPER, respectively (Fig. 3*A*). A greater V_T_ was denoted consistently in HYPER (*P* = 0.014).

Significant moderate correlations were detected between preapneic V_T_ and respectively $\text{P}{\text{ET}}_{{\text{O}}_{2}}$ (*r* = 0.76, *R*^2^ = 0.58, *P* < 0.001) and $\text{P}{\text{ET}}_{{\text{CO}}_{2}}$ (*r* = −0.57, *R*^2^ = 0.32, *P* < 0.001; Fig. 3, *B* and *C*).

#### Spleen and hemoglobin.

Splenic volume reductions were recorded in both apneic protocols (*P* = 0.019; Table 3). A stronger contraction was discerned during the first (29 ± 12% vs. 17 ± 10%, *P* < 0.001) and third (56 ± 9% vs. 49 ± 7%, *P* = 0.009) apneas in HYPER compared with NORM (Table 3).

**Table 3.** Breath-hold durations and corresponding physiological responses following each successive maximal static apneic attempt

	NORM				HYPER			
Variables	Baseline	1	2	3	Baseline	1	2	3
Time, s	–	154 ± 60	185 ± 61*	204 ± 62*	–	188 ± 43†	225 ± 45*†	239 ± 52*†
Time in *PhI*, s	–	19 ± 6	20 ± 5	22 ± 6	–	24 ± 6	24 ± 8	26 ± 5
Time in *PhII*, s	–	75 ± 29	110 ± 50*	119 ± 34*	–	109 ± 40†	147 ± 34*†	140 ± 23†
Time in *PhIII*, s	–	68 ± 33	65 ± 25	75 ± 30	–	59 ± 22	59 ± 20	78 ± 42
IBM, s	–	84 ± 28	98 ± 35*	112 ± 43*	–	128 ± 49†	146 ± 43*†	159 ± 46*†
HR, beats/min	71 ± 11	59 ± 15	60 ± 20	62 ± 18	67 ± 11	60 ± 20	64 ± 17	68 ± 15
SAP, mmHg	127 ± 10	177 ± 31*	194 ± 24*	208 ± 36*	131 ± 11	191 ± 20*	191 ± 33*	207 ± 27*
DAP, mmHg	79 ± 9	109 ± 12*	109 ± 17*	112 ± 15*	81 ± 6	109 ± 11*	117 ± 18*	115 ± 14*
MAP, mmHg	97 ± 11	138 ± 20*	143 ± 16*	149 ± 20*	100 ± 7	141 ± 13*	153 ± 20*	147 ± 10*
Stroke volume, mL/beat	81 ± 15	88 ± 28	89 ± 21	93 ± 21	80 ± 14	80 ± 20	73 ± 32	84 ± 24
Cardiac output, L/min	5.8 ± 1.7	4.7 ± 1.6	5.2 ± 1.9	5.7 ± 2.2	5.3 ± 1.3	4.7 ± 1.6	5.0 ± 2.6	5.2 ± 1.7
TPR, mmHg·min·L^−1^	18 ± 6	32 ± 12	30 ± 9*	31 ± 17	20 ± 6	33 ± 11	44 ± 29	32 ± 16
$\text{S}{\text{p}}_{{\text{O}}_{2}}$, %	98 ± 1	89 ± 7*	83 ± 11*	79 ± 11*	98 ± 1	84 ± 9*	77 ± 10*	73 ± 9*†
$\text{S}{\text{p}}_{{\text{O}}_{2}}$ nadir, %	98 ± 1	87 ± 7*	81 ± 11*	77 ± 12*	98 ± 1	82 ± 11*	73 ± 11*†	68 ± 9*†
Splenic volume, mL	267 ± 53	218 ± 40*	177 ± 35*	135 ± 44*	264 ± 42	184 ± 32*†	162 ± 23*	114 ± 38*†
Hemoglobin, g/L	150 ± 10	154 ± 14	–	157 ± 11	148 ± 9	157 ± 15	–	158 ± 11*

Data are means ± SD. Data were analyzed with a two-way repeated measures ANOVA, followed by Bonferroni post hoc test (*P* < 0.05). *Significant (*P* < 0.05) difference from baseline and in the absence of baseline from *apnea 1* (i.e., time, time in *PhI*, *PhII*, *PhIII*, IBM); †significant (*P* < 0.05) between protocol differences. DAP, diastolic arterial pressure; HR, heart rate; HYPER, hyperventilation; IBM, involuntary breathing movements; MAP, mean arterial pressure; NORM, normal; *PhI*, *phase 1*; *PhII*, *phase 2*; *PhIII*, *phase 2*; SAP, systolic arterial pressure; $\text{S}{\text{p}}_{{\text{O}}_{2}}$, peripheral oxyhemoglobin saturation.

Hemoglobin was significantly elevated from basal levels following the third apneic repetition in HYPER (*P* = 0.049; Table 3), whereas no differences were identified in NORM (*P* ≥ 0.188) nor between protocols (*P* = 0.603).

## DISCUSSION

This study sought to evaluate the combined effects of fasting with or without hyperventilation on apnea-induced physiological responses over a series of repeated breath-holds. The primary findings were that, in a fasted state, preapnea hyperventilation compared with normal breathing led to significantly longer apneas, which were terminated at progressively lower $\text{P}{\text{ET}}_{{\text{O}}_{2}}$ levels. On several occasions during the HYPER session (i.e., 8 of 9), subjects reached our safety threshold ($\text{S}{\text{p}}_{{\text{O}}_{2}}$ ∼65%) and were asked to abort their apneic attempts, with the preponderance of these incidents (i.e., 6 of 9) occurring during the third repetition (1 in NORM). In agreement with our hypothesis, preapneic fasting combined with hyperventilation may increase the risk for a hypoxic blackout; a risk that is aggravated over a series of repeated maximal attempts.

**Figure 4.**
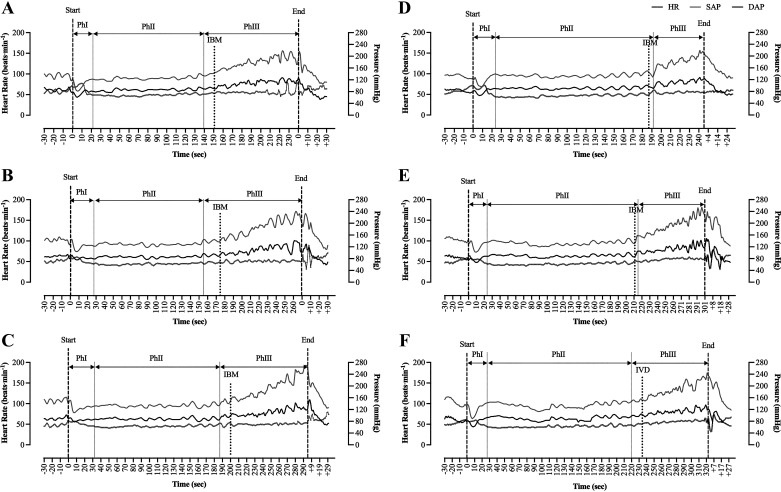
Beat-to-beat values of HR, SAP, and DAP during each successive maximal static attempt for NORM (*A–C*) and HYPER (*D–F*) in a representative subject. Apnea was divided into phases according to changes in cardiovascular variables: *phase I*, transition phase; *phase II*, “steady-state” phase; *phase III*, increases in SAP and DAP with slight changes in HR. Vertical dashed line, the start and volitional end of the apneic attempt; black vertical dotted line, first registered IBM; gray vertical dotted line, start of each phase. DAP, diastolic arterial pressure; HR, heart rate; HYPER, hyperventilation; IBM, involuntary breathing movement; NORM, normal breathing; SAP, systolic arterial pressure.

Upon the onset of each maximal attempt, we systematically recorded a transient fall in MAP coinciding with tachycardia (Fig. 4), modifications that were of similar magnitude across protocols and in line with those previously described in the literature (2, 27, 28). These hemodynamic adjustments are likely ascribed to an elevated intrathoracic pressure, brought on by the deep inspiration performed before each bout, as well as by the cessation of breathing, conjointly facilitating a reduction in SV and CO, concurrently lowering venous return (29–31). Thenceforth, a cardiovascular steady state was reached that spanned across the entirety of *PhII* and remained largely uninterrupted until the first IBM was registered and *PhIII* ensued (Fig. 4). In parallel with the diaphragmatic oscillations, we noted a continuous increase in arterial pressures, while CO was partially restored to preapneic levels. Interestingly, *PhII* was successively extended over the sequential attempts and lasted on average ∼31 s longer in HYPER (Table 3), whereas no differences were discerned neither in *PhI* nor *PhIII*. Therefore, our findings indicate a shift in the excitation of ventilatory sensory chemoreflexes, an assumption that is partly substantiated by the moderate correlation identified between the onset of IBMs and *PhIII* (*r* = 0.68, *R*^2^ = 0.47). Yet, in spite of these timing differences, collocation of the cardiovascular indices suggested a similar magnitude of response between the two respiratory conditions, both in *PhII* and *PhIII* (Table 3). Taken together, present findings signify that a 30-s hyperventilation period alters the apnea-induced hemodynamic responses in terms of onset time but not the magnitude of changes.

**Figure 5.**
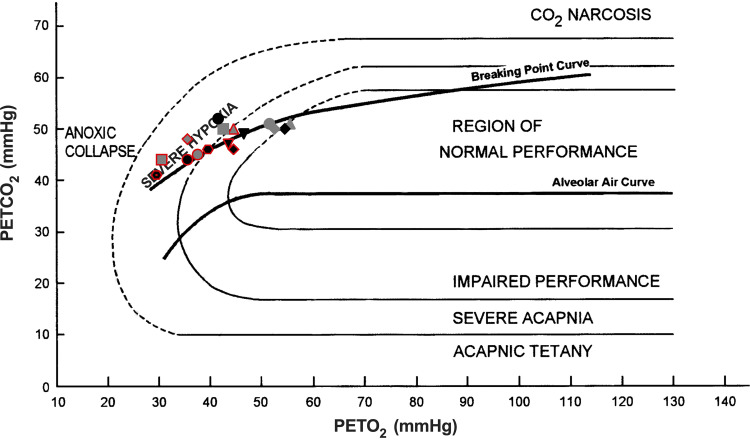
Oxygen/carbon dioxide nomogram (32) adapted from Lindholm and Gennser (33) depicting the end-tidal gas composition for apneas that were terminated at the safety threshold ($\text{S}{\text{p}}_{{\text{O}}_{2}}$ 65%; red outline symbols) matched with their corresponding apneas that were volitionally terminated at the conventional breaking point (symbol matched). One subject (hexagon symbol) reached the safety threshold during the third apnea in both HYPER (hexagon symbol, red outline, red dot) and NORM (hexagon symbol, red outline) protocols. The normal alveolar air (32) and the apnea breaking-point curves (34) are reported as well as the lines defining the regions on normal and impaired visual performance (32). HYPER, hyperventilation; NORM, normal breathing; $\text{P}{\text{ET}}_{{\text{O}}_{2}}$, partial end-tidal pressure of oxygen; $\text{P}{\text{ET}}_{{\text{CO}}_{2}}$, partial end-tidal pressure of carbon dioxide.

In HYPER, subjects attained progressively longer breath-holds than in NORM; hence, from a performance perspective, preapnea hyperventilation in a fasted state (i.e., compared with apneas in a fasted state preceded by normal breathing) conferred an advantage (Table 3; Fig. 2*A*). Conversely, it equally raised safety concerns given that significantly lower end-apneic $\text{S}{\text{p}}_{{\text{O}}_{2}}$ and $\text{P}{\text{ET}}_{{\text{O}}_{2}}$ levels were detected, when the hyperventilatory maneuver preceded (Tables 2 and 3). In this protocol, the subjects commenced their maximal attempts in a more pronounced hypocapnic state allied with a lipid-dominant metabolism. Thus, at the immediate onset of each apnea, the CO_2_ build-up started at a lower tension (Table 2), and thereon was associated with a slow accumulation rate. In tandem, delaying the physiological breaking point and enabling longer breath-holds to be reached. However, a risk imposed by these CO_2_ modifications is that loss of consciousness may ensue without forewarning as, by the time the urge to breathe rises, Po_2_ may fall below its critical level (10). Evidently, during HYPER, five of nine subjects (i.e., contrary to only 1 in NORM) reached our safety threshold ($\text{S}{\text{p}}_{{\text{O}}_{2}}$ 65%) and were asked to abort their attempts. Alarmingly, when questioned afterward, none of them could intuit how close they were to the blackout zone, with their end-apneic $\text{P}{\text{ET}}_{{\text{O}}_{2}}$ levels (5.1 ± 1.6 kPa; ∼ 38 ± 12 mmHg) ranging slightly above the theoretical limit of consciousness (∼3.6 kPa; ∼27 mmHg; Fig. 2*C*) (10) and falling within the severe hypoxia/impaired performance region (Fig. 5; 32). Given this cohorts’ profound reliance on hypercapnic stimuli in discerning their apneic endpoints (7, 17), it is perhaps not surprising that the preponderance of these events occurred in HYPER. Therefore, while fasting and hyperventilation improves apneic performance this combination may also increase the likelihood of sustaining a hypoxic blackout; a risk that is aggravated over serial attempts.

During both apneic interventions, we observed gradual shifts in preapneic gas tensions (Table 2; Fig. 2, *C* and *D*), shining light toward an under-conversed and often-overlooked risk associated with repeated bouts. Notably, preapneic levels of $\text{P}{\text{ET}}_{{\text{O}}_{2}}$ were progressively higher and $\text{P}{\text{ET}}_{{\text{CO}}_{2}}$ were successively lower than the preceding ones (Table 2; Fig. 2, *C* and *D*)—corroborating earlier reports (35). These responses may provide a plausible explanation behind the potentiated risk presented by repeated breath-holding. Conceivably, progressively lower CO_2_ tensions may gradually delay the excitation of ventilatory sensory chemoreflexes (36), making the apneist more susceptible to desaturation and increasing the risk of suffering a hypoxic blackout. In support of this notion is the fact that the majority of incidents necessitating our intercession (6 of 9) occurred during the final repetition. This is of key relevance since both the pre- and postapneic $\text{P}{\text{ET}}_{{\text{CO}}_{2}}$ levels were among the lowest registered across all trials (Table 2; Fig. 2*D*). In fact, in HYPER, wherein the highest proportion of incidents befell, the end-apneic $\text{P}{\text{ET}}_{{\text{CO}}_{2}}$ levels were on average ∼0.50 kPa lower than in NORM. It can thus be deduced that the hypercapnic input and, concomitantly, the respiratory drive was not intense enough to actuate the breaking point, thus exposing the apneist to critically low oxygen tensions. The question which then arises is: what is the underlying mechanism(s) governing such effects?

An intriguing finding was the subtle and continuous rise in preapneic V_T_ breathing detected across the serial bouts (Fig. 3*A*). This stepwise rise was apparent in both protocols, coinciding and correlating well with the preapneic end-tidal gas changes ($\text{P}{\text{ET}}_{{\text{O}}_{2}}$, *r* = 0.74; $\text{P}{\text{ET}}_{{\text{CO}}_{2}}$, *r* = −0.52; Fig. 3, *B* and *C*). It is currently a common practice among breath-hold divers to perform a series of ribcage and diaphragmatic stretching drills before engaging in apneic activities (personal communications/observations). Notably, respiratory muscle stretching has been shown to transiently improve V_T_ breathing, with documented mean increases of 120 mL (37). Considering that, at least in HYPER, the *f*_B_ was kept constant before each attempt, then the V_T_ changes should have emanated from an elevated lung compliance and/or a relaxed airway pressure, facilitated by the diaphragmatic oscillations. These adjustments would probably have reduced the inspiratory effort per liter of air inspired, enabling deeper breathing movements to be attained, leading to apneas commencing at higher $\text{P}{\text{ET}}_{{\text{O}}_{2}}$ and lower $\text{P}{\text{ET}}_{{\text{CO}}_{2}}$. However, regardless of the exact underlying mechanism(s), this novel finding demonstrates that even if *f*_B_ is maintained stable and hyperventilation is intended to be avoided, over serial attempts, unintentional increases in V_T_ may insidiously predispose the apneist to a more pronounced arterial desaturation. Accordingly, raising awareness concerning this phenomenon is imperative from a safety standpoint.

Splenic contractions were evident across the sequential attempts and protocols (Table 3). These reductions were intensified over the serial bouts and were greater during the final repetition in HYPER (Table 3). Considering that hypoxia dictates, in a dose-dependent manner, the magnitude of the splenic response (17, 38), present findings are likely related both to the degree and duration of hypoxemic stress experienced by the apneist during HYPER. As a result, these contractions led to marginal increases in hemoglobin concentrations (Table 3). Such elevations could, theoretically, improve the oxygen binding and carrying capacity of blood (39, 40); hence, increasing the oxygen reserve by the systemic mobilization of erythrocytes. Thereon, successive apneas could commence with a greater amount of readily available oxygen. It is thus, plausible that the splenic response may also have contributed toward the preapneic gas tension shifts and the performance increments reported across the serial attempts.

The proportion of subjects that reached our termination criteria is, to the best of our knowledge, the highest reported in the literature, bearing testament to the danger posed by HYPER (16, 17, 41). It is also important to highlight here that in this study we used an experimental design composing of dry static apneas, a modality commonly associated with a less severe hypoxemic stress than, for instance, dynamic apneas (25, 39). Although present findings should be interpreted within the context they have been examined (i.e., dry static apneas, nondiving cohort), it does beg the question as to how much more common such incidents may be if the present intervention was used in conjunction with muscular exercise (11, 12, 33). In this context, evidence suggest that apneic activities incorporating an exercise component are associated with a higher prevalence of hypoxic blackouts than static apneas (20, 39). Therefore, apneists utilizing fasting and hyperventilation as part of their preparatory routines must err on the side of caution, especially if they plan to perform a series of repeated attempts, whether that be of static and/or dynamic nature.

In conclusion, this study demonstrates that apneas preceded by a 12-h overnight fast coupled with a 30-s hyperventilation as opposed to normal breathing may exacerbate the risk of a hypoxic blackout. This risk is evidently potentiated over a series of repeated apneas, possibly due to a gradual rise in V_T_ breathing, concurrently further reducing preapneic CO_2_ tensions, delaying the physiological breaking point and, resultantly, exposing apneist to a greater degree of hypoxemia.

### Perspectives and Significance

This study shows that breath-holds preceded by a 12-h overnight fast coupled with a 30-s hyperventilation as opposed to normal breathing may increase the likelihood of a hypoxic blackout through delaying the excitation of hypercapnic ventilatory sensory chemoreflexes – a risk that is exacerbated over a series of repeated maximal apneic bouts. Interestingly, across both protocols, even if the *f*_B_ is maintained stable and hyperventilation is intended to be avoided, over serial attempts, unintentional increases in V_T_ breathing may insidiously predispose the apneist to a more pronounced arterial desaturation. In view of the present findings apneists utilizing fasting in isolation or in combination with hyperventilation as part of their preparatory routines must err on the side of caution, especially if they plan to perform a series of repeated attempts.

### Experimental Considerations

In the past, the effects of fasting states (16, 17) and hyperventilation (35, 41) on repeated apneic performance and associated physiological responses have been assessed in isolation. These studies highlighted that both fasting and hyperventilation alone increase susceptibility to hypoxic blackout (16, 17). Therefore, the present study was specifically designed to explore the synergistic effects of fasting with or without preapneic hyperventilation to evaluate whether this combination would further exacerbate the risks posed by fasting in isolation. In this regard, a retrospective analysis of our data with those where hyperventilation was used without an overnight fast (35, 41) highlight that combining these risk factors further increases the likelihood of sustaining a hypoxic blackout. Yet, an experimental session where hyperventilation was also performed under a fed-state would certainly have provided additional insights to the magnitude of this response.

## DATA AVAILABILITY

The datasets presented in this article are not readily available as sharing these will compromise the ethical standards and agreement with the subjects.

## GRANTS

This study was supported by the Swedish Defense Forces Grant 9220919. M.E.K. was supported by a salary grant from the KTH Royal Institute of Technology (Grant: C-2020-0748).

## DISCLOSURES

No conflicts of interest, financial or otherwise, are declared by the authors.

## AUTHOR CONTRIBUTIONS

A.E. conceived research; A.E. and M.G. designed research; A.E., M.G., O.E., and M.E.K. performed experiments; A.E. and M.E.K. analyzed data; A.E. interpreted results of experiments; A.E. prepared figures; A.E. drafted manuscript; A.E., M.G., O.E., and M.E.K. edited and revised manuscript; A.E., M.G., O.E., and M.E.K. approved final version of manuscript.
